# Complex interactions of *Tyrp1* in the eye

**Published:** 2011-09-22

**Authors:** Hong Lu, Liyuan Li, Edmond R. Watson, Robert W. Williams, Eldon E. Geisert, Monica M. Jablonski, Lu Lu

**Affiliations:** 1 Department of Ophthalmology, Hamilton Eye Institute, University of Tennessee Health Science Center, Memphis, TN; 2 Department of Ophthalmology, Affiliated Hospital of Nantong University, Nantong, China; 3 Department of Anatomy and Neurobiology, University of Tennessee Health Science Center, Memphis, TN; 4 Jiangsu Key Laboratory of Neuroregeneration, Nantong University, Nantong, China

## Abstract

**Purpose:**

To use a systems genetics approach to construct and analyze co-expression networks that are causally linked to mutations in a key pigementation gene, tyrosinase-related protein 1 (*Tyrp1*), that is associated both with oculocutaneous albinism type 3 (OCA3) in humans and with glaucoma in mice.

**Methods:**

Gene expression patterns were measured in whole eyes of a large family of BXD recombinant inbred (RI) mice derived from parental lines that encode for wildtype (C57BL/6J) and mutant (DBA/2J) *Tyrp1*. Protein levels of Tyrp1 were measured in whole eyes and isolated irides. Bioinformatics analyses were performed on the expression data along with our archived sequence data. Separate data sets were generated which were comprised of strains that harbor either wildtype or mutant *Tyrp1* and each was mined individually to identify gene networks that covary significantly with each isoform of *Tyrp1*. Ontology trees and network graphs were generated to probe essential function and statistical significance of covariation. Genes with strong covariance in wildtype mice were assembled into genome-wide heatmaps for cohorts carrying either wildtype or mutant *Tyrp1*.

**Results:**

Single nucleotide polymorphism (SNP) analysis verified the presence of the Tyrp1b mutation in the Tyrp1 gene. Message levels were greater in BXD strains with the mutant Tyrp1. Interval mapping of these BXD mice revealed a strong expression quantitative trait locus (eQTL) on Chr 4 at the location of the gene itself. Composite mapping revealed a suggestive eQTL on Chr 9 at the location of myosin-Va (Myo5a), mutations in which are known as dilute. Enriched biologic processes associated with wildtype *Tyrp1* included pigmentation, melanin biosynthetic process, and mesenchymal cell development, while associations with the mutant gene included categories of neural crest cell development, protein metabolic processes and glycoprotein metabolic processes. Genome-wide heatmaps revealed strong candidate cis-eQTLs on Chr 4 at *Tyrp1* and on Chr 9 at *Myo5a* in all mice. In the wildtype data set, *Tyrp1* was an upstream regulator of six pigmentation and two mesenchyme genes. In addition, five genes, including *Tyrp1,* were at least partially regulated by *Myo5a*. Analyses of the strains harboring the mutant gene revealed significant loss of correlation to traditional genes and gain of correlation to genes with little or no functional relationship.

**Conclusions:**

These findings indicate that the *Tyrp1^b^* mutation modifies the pathways and gene networks in which *Tyrp1* functions. Our results also indicate direct and indirect regulatory control of *Tyrp1* and other pigmentation and mesenchymal genes by *Myo5a*. Lastly, we find that the mutations reduce the ability of *Tyrp1* to regulate expression of other genes that participate in pigmentation metabolism.

## Introduction

*TYRP1* (tyrosinase-related protein-1) is a melanosome-specific gene [1] that is involved in pigment synthesis. In humans, mutations in this gene cause oculocutaneous albinism type 3 (OCA3) in an autosomal recessive inheritance pattern [2]. OCA3 is present in blacks of southern African descent [3] at a frequency of ~1:8,500 [2]. Mutations in *TYRP1* have also been found in individuals of Caucasian German [4], Asian Indian [5], and Pakistani descent [6]. The clinical manifestations of OCA3 include copper-red coloration of the skin and hair along with dilution of the iris color, nystagmus and/or strabismus, photophobia and visual impairment [2,7,8].

Pigment synthesis takes place in the melanosome, a lipid-bound organelle within melanocytes. The production of brown-black pigment or eumelanin is a multistep chemical reaction that is regulated by multiple gene products including tyrosinase-related proteins—TYRP1, TYRP2 (dopachrome tautomerase, DCT) and tyrosinase (TYR; reviewed in [9]). This complex process begins with the rate-limiting catalysis of tyrosine or dopa to dopaquinone by the enzyme TYR. DCT is responsible for the catalysis of dopachrome to the 5,6-dihydroxyindole 2-carboxylic acid (DHICA) intermediate. Subsequently DHICA is catalyzed by TYRP1 to 5,6-indolequinone-2-carboxylic acid, which is then incorporated into eumelanin. Other gene products such as silver (SI), another melanosome protein, microphthalmia-associated transcription factor (MITF), a signal protein, and myosin-Va (MYO5A), a motor protein, are also critical for melanogenesis.

In mice, *Tyrp1* is also known as the *brown* coat color locus [10]. Although the *brown* allele harbors two missense nucleotide substitutions (i.e., G→A at nucleotide 598 and G→A at nucleotide 1246), the elegant studies of Jackson and colleagues [11] demonstrate that only the former is the *Tyrp1^b^* mutation. The functional cause for the brown coat color is the inability of mutant TYRP1 to catalyze the synthesis of 5,6-indolequinone-2-carboxylic acid, yielding brown rather than black eumelanin [12]. Digenic mutations in *Tyrp1* and Glycoprotein non-metastatic melanoma protein B (*Gpnmb*) cause pigmentary dispersion syndrome and pigmentary glaucoma in DBA/2J mice [13]. While no exonic mutations in either gene have been found in human glaucoma patients [13,14], given the data for mice, one would anticipate that only a “two-hit” model would be required to reveal the coupling to disease in humans.

Recombinant inbred (RI) strains of mice allow us to identify the genetic basis for variation in phenotype, in this case, the expression of *Tyrp1* and the network in which it functions. The largest panel of RI strains—the BXD family—consists of the inbred progeny of a cross between C57BL/6J (B6 or B) that has no aberrant ocular phenotype and DBA/2J (D2 or D) that harbor mutations in *Tyrp1* and *Gpnmb*. Collectively, these 80+ BXD lines have been used extensively in genetic and genomic studies of the eye and central visual system [15-20]. Because one parent has wildtype alleles of *Tyrp1* while the other carries the mutant alleles, we can exploit the segregation of the mutation in the RI lines to compare and contrast expression networks associated with *Tyrp1*.

The purpose of this investigation was to use a systems genetics approach to construct co-expression networks and determine the genetic regulation of both wildtype and mutant *Tyrp1*. Using expression quantitative trait locus (eQTL)  mapping, we were able to identify candidate cis- and trans-eQTLs that modulate *Tyrp1* expression levels. Additionally, by segregating strains based upon the presence or absence of the *Tyrp1^b^* mutation, we were able to construct distinct co-expression networks of genes that are associated with the mutant and wildtype genes. Further, we identified potential upstream modulators and downstream genes that are affected by *Tyrp1*.

## Methods

### BXD strains, generation of microarray data and western blotting

All experimental protocols were approved by the Animal Care and Use review board of the University of Tennessee Health Science Center. Mice were handled in a manner consistent with the ARVO Statement for the Use of Animals in Ophthalmic and Vision Research and the Guide for the Care and Use of Laboratory Animals (Institute of Laboratory Animal Resources, the Public Health Service Policy on Humane Care and Use of Laboratory Animals). A total of 292 adult mice of both sexes were used for this study: 16 mice from parental strains (four male and four female of each parent strain), eight mice from F1 hybrid strains (two male and two female from both F1 hybrid matings), and 268 BXD RI mice (two male and two female from each of 67 BXD strains). Animals were maintained per our published protocols [17,18].

RNA isolation, microarray hybridization, and microarray data normalization were performed using our published protocols [17,18]. RNA isolated from eyes of BXD strains, their parental strains, and F1 hybrids were hybridized to Affymetrix microarrays (M430V2; Affymetrix, Santa Clara, CA). Two arrays were prepared and analyzed for each of the strains and consisted of four eyes from both male and female mice. Averages were taken for strains carrying either wildtype or mutant *Tyrp1* and an unpaired *t*-test was performed to determine if there were significant differences in expression levels between the two groups.

Western blots were performed using our standard protocols [17]. Eyes (n=2 for each parent strain; one eye per mouse) were debrided of extraocular tissue and the optic nerve was clipped as close to the globe as possible. Irides samples (n=2 from each parent strain; two irides pooled per sample) were carefully dissected from the eye after removal of the anterior segment. Total protein (10 μl) was loaded into each well of a 4%–12% NuPAGE gel (Invitrogen, Carlsbad, CA). A polyclonal anti-Tyrp1 antibody was generously provided by Dr. Vincent Hearing at the National Cancer Institute. Anti-glyceraldehyde 3-phosphate dehydrogenase (Gapdh; Cell Signaling Technology, Danver, MA) was used to normalize protein loading across all wells. Blots were scanned on a Kodak Image Station 4000MM (Eastman Kodak Co., Rochester, NY) and the intensity of each immunopositive band was quantified using Molecular Imaging Software (version 4.5.1; Eastman Kodak Company). Unpaired *t*-tests were performed to determine if significant differences in protein levels were present between samples derived from either whole eye or iris samples.

### Expression QTL (eQTL) mapping, SNP analysis, and heritability calculation

On the Affymetrix 430V2 array, *Tyrp1* is represented by three probe sets – 1415861_at, 1415862_at, and 1439409_x_at. Each probe set varied in expression level, hybridization location, and cis-eQTL significance level. When considering these factors for each probe set, 1415862_at had the most relevant relationship with the *Tyrp1* gene – this probe hybridized to the last 3 exons and proximal 3′ UTR (located at Chromosome 4, 80.49256) of *Tyrp1*, had the highest likelihood ratio statistics (LRS) score for its cis-eQTL and had a significant expression level of 12.798. For these reasons, the 1415862_at probe set was used as the single representation of *Tyrp1* expression in these studies.

QTL mapping was performed using the WebQTL module on GeneNetwork using our published methods [15,18,21]. As in our previous study [17], BXD24 was excluded from this investigation because this line has retinal degeneration due to a spontaneous mutation in centrosomal protein 290kDa (Cep290) [22]. Simple interval mapping was performed to illustrate the significance of any eQTLs that regulate *Tyrp1* expression. Significance levels were estimated by permutation analyses. Composite interval mapping was also performed to control for genetic variance associated with major eQTLs and therefore identify any secondary eQTLs that may have been otherwise masked. Each of these analyses produce a likelihood ratio statistic (LRS) score, providing us with a quantitative measure of confidence of linkage between the observed phenotype—in this case variation in expression level of *Tyrp1*—and SNP markers.

Sequence variability between B6 and D2 were determined using the single nucleotide polymorphism (SNP) variant browser link on GeneNetwork. Heritability of the expression level of *Tyrp1* was calculated using the formula of Hegmann and Possidente [23]: *h* ^2^=0.5Vg / (0.5Vg + Ve) where h^2^ is the heritability, Vg is the genetic variance and Ve is the environmental variance. The factor of 0.5 in this ratio was applied to adjust for the twofold increase of additive genetic variance among inbred strains relative to outbred populations [15,17].

### Correlation and heatmap analyses, gene ontology tree machine (GOTM), and gene network construction

The wildtype and mutant data sets were analyzed as a large combined data set, as well as individual separate data sets. This allowed us to directly assess the influence of *Tyrp1* mutations on global expression levels in the eyes and any resulting modifications of co-regulatory networks. Within a data set generated with RI strains having either wildtype or mutant *Tyrp1*, the expression level of *Tyrp1* was compared to 45,101 probes present on the M430v2 array and the top 500 genetically correlated genes were selected. Correlative analysis was calculated using Spearman’s rank correlations, which was computed using tools on GeneNetwork.

After removing Riken clones, intergenic sequences, predicted genes, and probes not associated with functional mouse genes, the remaining list of correlates with mean expression levels above baseline in the eye were analyzed by Gene Ontology (GO) enrichment analysis [24] via a link on GeneNetwork. Enriched GO terms were visualized as directed acyclic graphs (DAGs). The p values generated from the hypergeometric test were automatically adjusted to account for multiple comparisons using the Benjamini and Hochberg correction [25]. Each node in the DAG is a separate GO category. GO categories that are outlined in bold and list an adjusted p value of <0.05 are the enriched GO categories that are statistically over-represented in the set of genes that were submitted for analysis. Categories outlined by thin black lines are non-enriched parent categories. Each node shows the name of the GO category, number of genes in the category and the adjusted *p* value indicating the significance of enrichment.

Spring Model Layout Network Graphs were constructed using the genes contained within the enriched GO categories in the DAGs. Each node in a graph represents an individual transcript and interconnecting lines illustrate ranges of Pearson correlation coefficient values.

### Heatmap construction and partial correlation analyses

Using WebGestalt via links in GeneNetwork [26,27], heatmaps were constructed using the genes from statistically significant GO categories. Heatmaps were constructed using all three data sets: combined data set; strains that carry wildtype *Tyrp1*; and strains that carry mutant *Tyrp1*. Each of these three data sets contained the 17 genes that were present in the enriched GO categories generated from strains that have wildtype *Tyrp1*. The purpose of using only this selected subset of genes was to highlight potential upstream and downstream regulation of wildtype *Tyrp1* and to determine if relationships were disrupted in mutants. The method of partial correlation implemented in GeneNetwork [28,29] was also used to test the causal involvement of *Tyrp1* on covariation between *Myo5a* and other putative targets identified in heatmaps.

## Results

### Tyrp1 expression levels in eyes of BXD mice

**Figure 1:**
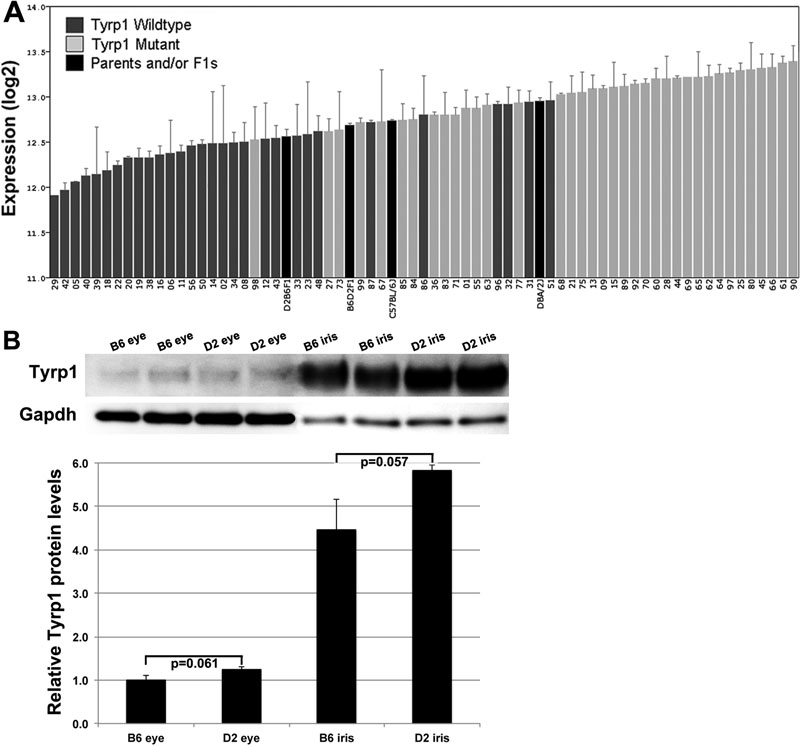
Expression levels of *Tyrp1* across strains. **A**: Rank ordered mean of *Tyrp1* mRNA expression levels across 67 BXD RI strains, their parental strains and F1 crosses. Values denote normalized relative expression levels on a log2 scale (mean±SEM). The majority of lines with the *Tyrp1^b^* mutation expressed higher levels of *Tyrp1* than those with the wildtype allele. F1 and parental strains had intermediate mRNA expression levels. **B**: western blot showing Tyrp1 protein levels in eyes and irides of parental strains. Tyrp1 protein levels were normalized to Gapdh and the amount of protein in eyes of B6 mice was set to 1. There is no statistical difference in Tyrp1 protein levels between strains with wildtype versus mutant *Tyrp1*.

There was a high level of variation in the expression level of *Tyrp1* among the BXD strains (Figure 1). The average expression level of *Tyrp1* in all BXD strains was 12.8±0.1 (mean±SEM). The range included a high of 13.4±0.2 for BXD90 and a low of 11.9±0.1 for BXD29 (Figure 1A). In the B6 parental strain, *Tyrp1* had an average expression level of 12.7±0.1, which was significantly less than that of D2 parental strain (12.9±0.1; p=0.045). Similarly, lines with the *Tyrp1^b^* mutation had on average higher *Tyrp1* expression levels while those with wildtype gene had lower expression levels (13.0±0.1 and 12.5±0.1, respectively; p<0.0001).

Protein levels of Tyrp1 paralleled mRNA levels. Tyrp1 protein in whole eyes of the D2 strain was 1.25 old greater than that in B6, although this difference did not reach statistical significance (Figure 1B, p=0.06). Because mutations in *Tyrp1* cause iris atrophy in D2 mice [25], we determined the relative amounts of Tyrp1 protein in the iris of parental strains. Similar to the trend found in the whole eye, Tyrp1 protein was slightly more abundant in the iris of D2 than in B6 (p=0.057), although the difference was not statistically significant.

### eQTL mapping, SNPs and heritability calculation for control of *Tyrp1* expression levels

**Figure 2:**
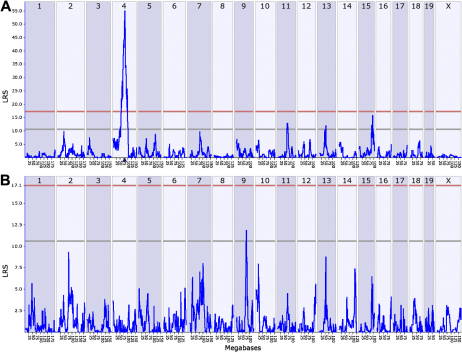
Graphic illustration of simple and composite mapping of *Tyrp1* expression in whole eyes. **A**: A significant eQTL is present at the location of *Tyrp1* on Chr 4, making it a candidate cis-eQTL. **B**: Composite interval mapping reveals a suggestive eQTL on chromosome 9 at the location of *Myo5a*. The blue traces in these interval maps indicate LRS scores across the genome. Horizontal lines mark the transcript-specific significant (LRS 17) and suggestive (LRS 11) thresholds based on results of 1,000 permutations of original trait data.

By performing simple interval mapping for *Tyrp1* (probe set 1415862_at), we found a highly significant eQTL with an LRS of 56 at the location of the gene itself (Figure 2A). As customary in QTL-based analyses, a cis-eQTL maps the source of variation in gene expression to a locus at or near the position of the gene itself. In contrast, a trans-eQTL maps the source of variation to an area of the genome away from the position of the gene. Composite interval mapping that mathematically controlled for the cis-eQTL on Chr 4 exposed a secondary trans-eQTL on Chr 9 at approximately 74–77 Mb with an LRS score of ~12.0 (Figure 2B). This location corresponds to the genomic location of *Myo5a* at 75.0 Mb. *Myo5a* is also known as the *dilute* locus and encodes a motor protein critical for melanin synthesis that is mutated in the D2 [30] and roughly half of all BXD strains. *Myo5a* is itself associated with a massive cis-eQTL (LRS of 150 – 160) with 3–4 fold higher expression in wildtype strains than in BXD strains that inherit the mutant *dilute* allele (data not shown).

There are two known missense exonic SNPs in *Tyrp1* of BXD strains with the D2 genotype [31]. We confirmed these and found an additional pair of synonymous exonic SNPs and many intronic SNPs using new open access sequence data resources at GeneNetwork and the Sanger Institute. A complete list of all SNPs is provided in Appendix 1. Heritability of *Tyrp1* expression levels was calculated on the separate wildtype and mutant data sets and the heritability was 0.25 and 0.33, respectively.

### Biologic process enrichment and co-expression networks

**Figure 3:**
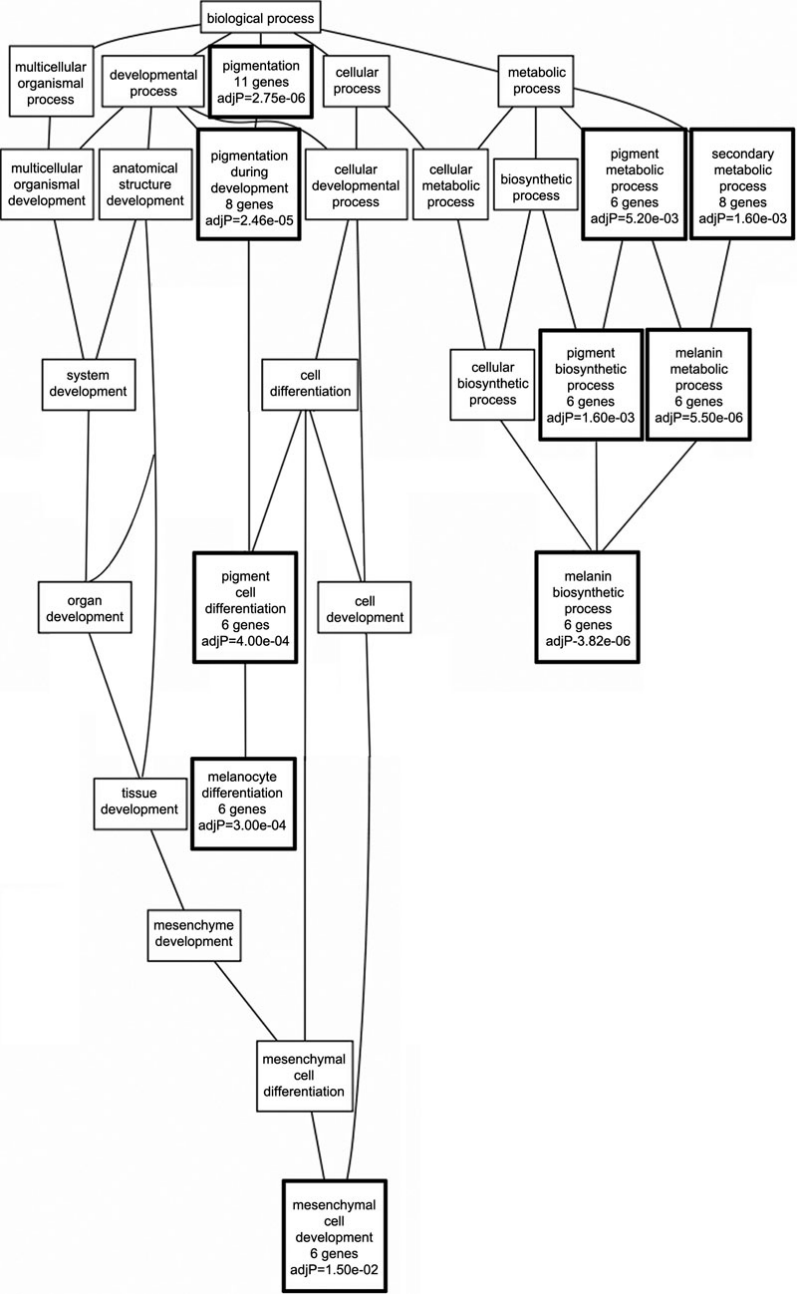
Genetic associations with wildtype *Tyrp1*. In BXD RI mice with wildtype *Tyrp1*, GO enrichment analysis illustrates that the majority of biologic processes to which transcripts correlated with *Tyrp1* expression belong include pigmentation, melanin metabolic process, pigment biosynthetic process, melanocyte differentiation and mesenchymal cell development. GO categories reaching statistical significance are indicated with a bold outline and adjusted p values.

Of the 500 transcripts with highest genetic correlation with wildtype *Tyrp1,* 368 remained after filtering. This list of genes was submitted for GO enrichment analysis and a directed acyclic graph (DAG) that grouped genes of similar biologic function was produced (Figure 3). The three main branches of the GO tree were related to melanocyte differentiation, melanin biosynthesis, and mesenchymal cell development. Ten of the 27 (37%) categories in the graph were statistically significant for enriched transcripts. Individual significant categories included “pigmentation” (11 genes), “pigment cell differentiation”(6 genes), “melanocyte differentiation” (6 genes), “melanin biosynthetic process” (6 genes), and “mesenchymal cell development” (6 genes). Of these 368 genes, only 17 unique transcripts were present in the GO categories that were statistically over-represented in the set of genes that was submitted. A list of the genes contained in each statistically significant category can be found in Appendix 2.

**Figure 4:**
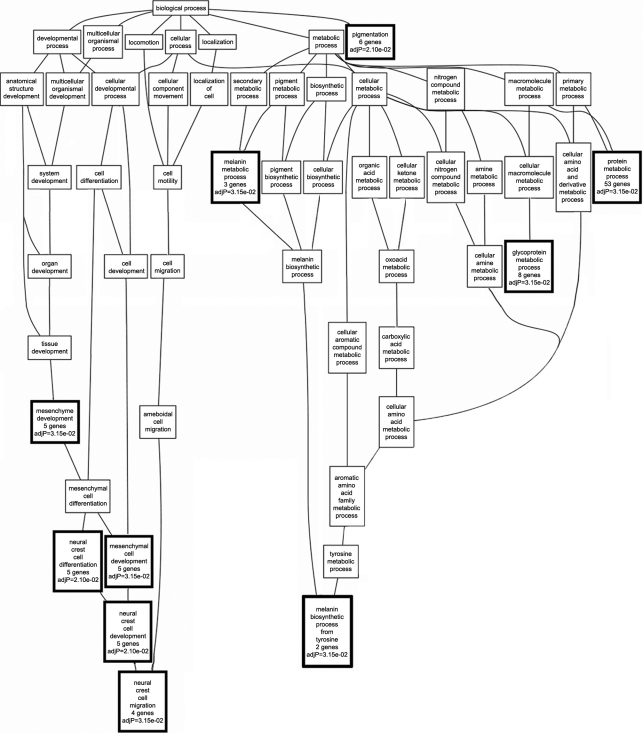
Genetic associations with mutant *Tyrp1*. In BXD RI mice with mutant *Tyrp1,* gene ontology enrichment analysis identified two distinct clusters of biologic processes that were correlated with mutant *Tyrp1*. The first branch included mesenchyme development and neural crest cell development and migration. The second branch included melanin biosynthetic processes and glycoprotein metabolic processes. GO categories reaching statistical significance are indicated with a bold outline and adjusted p values.

A GO tree was also generated from the 338 transcripts that remained after filtering the data set generated from the RI strains that carry mutant *Tyrp1* (Figure 4). Ten of the 53 (19%) categories in the graph were statistically over-represented in the set of 338 genes. The tree contained two branches related to neural crest cells and melanin synthesis. The first branch contained five categories that were statistically over-represented including “mesenchyme development” (5 genes) and “neural crest cell development” (5 genes). The second branch contained five unrelated statistically significant categories including “pigmentation” (6 genes), “melanin metabolic process” (3 genes), “glycoprotein metabolic process” (8 genes) and “protein metabolic process” (53 significant genes). Three significant categories, “melanin metabolic process,” “pigmentation,” and “mesenchymal cell development,” were present in GO trees generated from both wildtype and mutant GO data sets. A total of 64 non-redundant transcripts were present in the 10 statistically significant categories. A list of the genes contained in each significant category can be found in Appendix 3. Only six genes—dopachrome tautomerase (Dct), endothelin receptor type B (Ednrb), retinol dehydrogenase 10 (all-trans; Rdh10), semaphorin 3C (Sema3c), *Tyr* and *Tyrp1*—were shared in the lists of genes in the over-represented biologic process categories that were generated using both wildtype and mutant data sets. This indicates that the *Tyrp1^b^* mutation dramatically disrupts the cohort of genes with which *Tyrp1* interacts—only 9% of the genes are found in networks common to both the wildtype and mutant data sets.

**Figure 5:**
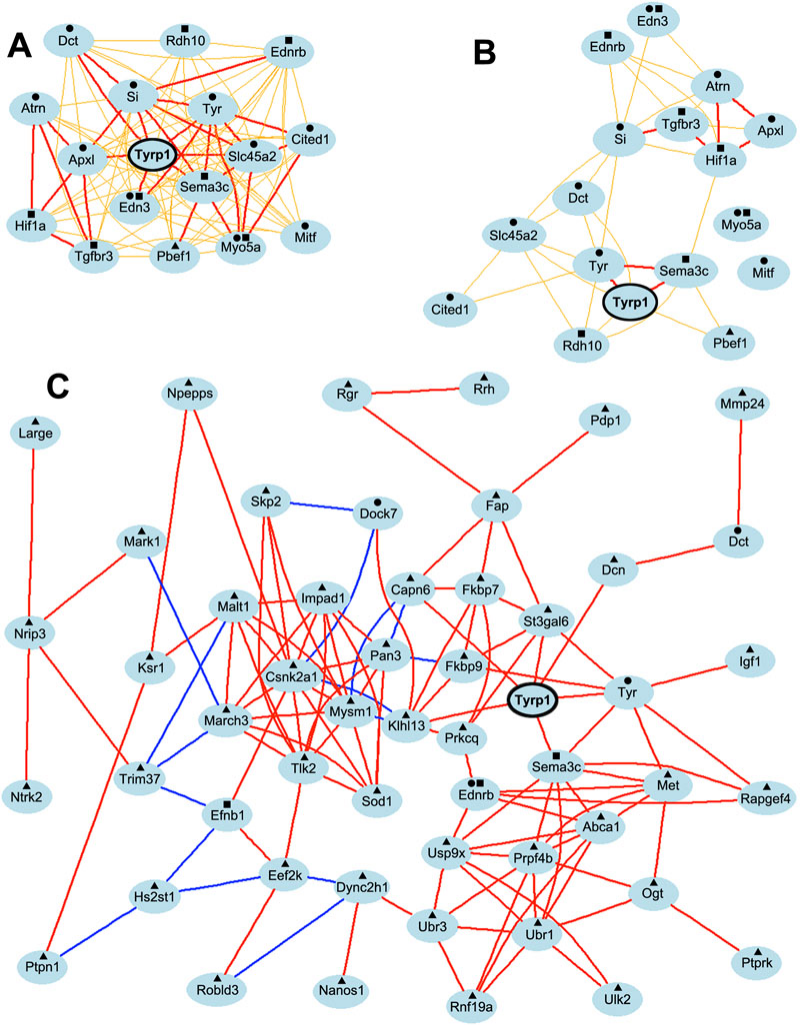
Genetic network co-expression graphs. **A**: Genetic coexpression network generated from genes correlated with wildtype *Tyrp1* shows that the expression level of *Tyrp1* was directly linked to all nodes in the network with an r value of ≥0.5. Moreover 94% of the genes function in mesenchyme development and/or melanin/pigment production. **B**: A similar network generated using the same 17 genes and expression levels from the mutant database indicates that the expression of *Tyrp1* is correlated with only six genes in the network and the tight cohesive nature of the network is lost. **C**: Genetic coexpression network generated from genes correlated with mutant *Tyrp1* shows that the expression level of mutant *Tyrp1* was directly linked to only six nodes at r≥0.7. The remaining associations occurred through intermediate links. Only 8% of the genes in the network function in melanin/pigmentation pathways and only 6% function in mesenchyme development, the two top biologic function categories associated with wildtype *Tyrp1*. ●=melanin/pigmentation pathway; ■=mesenchyme development; ▲=other biologic function. Each transcript is shown as a node and Pearson correlation coefficient values are indicated as lines between nodes. Bold and thin lines reflect coefficients of 1.0–0.7 and 0.7–0.5 respectively. Red/orange and blue/green colors indicate positive and negative correlation coefficients, respectively.

To graphically evaluate the strength of the Pearson correlation coefficients among genes with which *Tyrp1* is correlated, we generated three network graphs containing: the 17 transcripts from statistically significant categories from the wildtype data set; the same 17 transcripts generated from the wildtype data set using correlations derived from the mutant data set; and the 64 transcripts from statistically significant GO categories from the mutant data set. The graph generated with the transcripts from the wildtype data set showed a *Tyrp1*-centered graph with direct correlation of all genes to *Tyrp1* (Figure 5A). A high correlation coefficient (above 0.7) was present between *Tyrp1* and seven (44%) transcripts—silver (*Si*), *Sema3c*, endothelin 3 (Edn3), apical protein, Xenopus laevis-like (Apxl), Tyr, solute carrier family 45, member 2 (Slc45a2), and *Dct.* Importantly, several other clusters of highly correlated transcripts were also found among other genes in the network. These genes are included in all ten enriched GO categories of the DAG. This indicates that all genes in the network are tightly interlinked with each other and are found in multiple GO categories related to mesenchyme or melanin/pigmentation.

This same list of 17 genes was subjected to network analysis using the mutant data set. The network graph yielded an intermediate (r>0.5) correlation coefficient between *Tyrp1* and only two transcripts (13%). All other correlations were reduced to r values of between 0.5 and 0.7. Two transcripts—*Myo5a* and *Mitf*—had correlation values of <0.5 with all other transcripts in the network. Only six correlations (38%) with *Tyrp1* were direct. A small cluster of highly correlated transcripts was present—ataxia telangiectasia serine-protein kinase (*Atm*), transforming growth factor, beta receptor 3 (*Tgfbr3*), *Hif1a*, and *Apx1*. This same cluster was present in the network generated from the wildtype data set.

The network generated using the 64 transcripts from the mutant data set formed a fragmented graph comprised of 53 transcripts with Pearson correlation coefficients greater than 0.7. Moreover, *Tyrp1* was no longer the focal point of the network (Figure 5C). Six of 52 (11%) correlations with *Tyrp1*—*Tyr, Sema3c*, ST3 beta-galactoside alpha-2,3-sialyltransferase 6 (St3gal6), decorin (Dcn), calpain 6 (Capn6), and kelch-like 13 (Drosophila; *Khl13*) —were direct, whereas all other correlations with *Tyrp1* occurred via intermediaries. Only five of the 53 genes were also in the wildtype network. While several genes relevant to mesenchyme or melanin/pigmentation were present—*Dct*, dedicator of cytokinesis 7 (*Dock7*), ephrin B1 (*Ednrb*), *Efnb1*, *Sema3c* and *Tyr*—most (87%) of the genes functioned in other non-related pathways.

### Global Heatmap analyses

**Figure 6:**
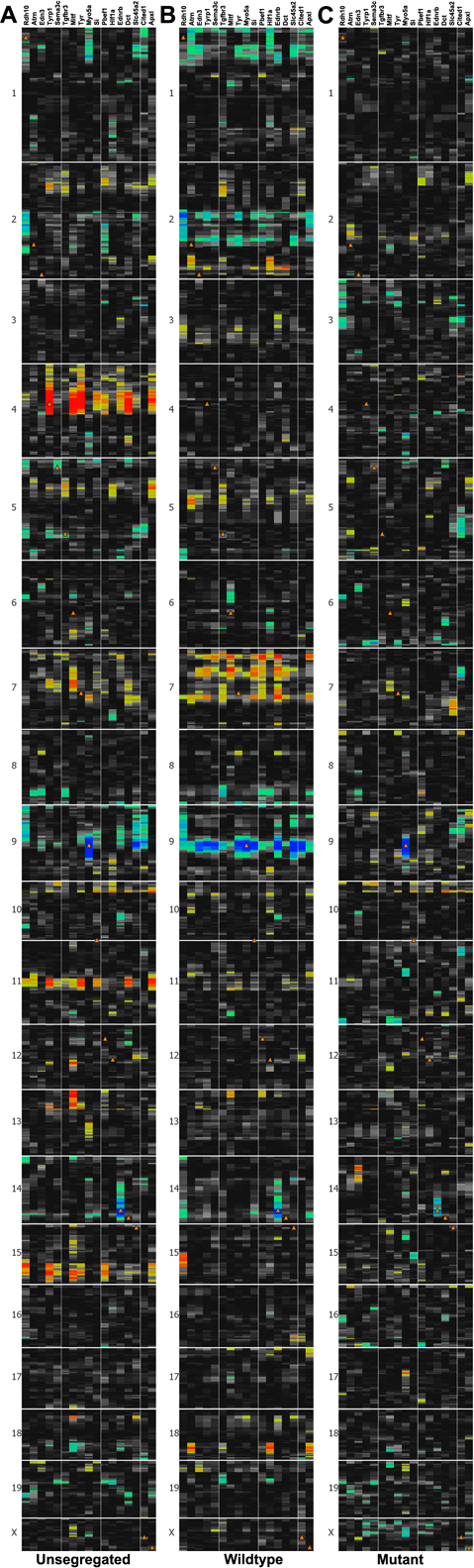
Heatmaps of *Tyrp1* covariates taken from the GO categories achieving statistical significance in the wildtype data set. **A**: In the combined data set, both *Tyrp1* and *Myo5a* are cis-eQTLs on chromosome 4 and 9, respectively. The red and blue bands at these same locations indicate which other genes are likely regulated by *Tyrp1* and *Myo5a*. **B**: In the data set comprised of RI lines with wildtype *Tyrp1*, the band on chromosome 4 is not present because the expression level of *Tyrp1* in these lines does not vary enough to be mapped. However, the band on chromosome 9 remains and is enlarged, suggesting that Myo5a influences the expression of multiple genes in the heatmap. **C**: In the data set comprised of lines with mutant *Tyrp1*, *Myo5a* remains as a cis-eQTL but all other associations are absent. The orange triangles indicate where each gene is located in the genome. Red or blue bars indicate where each gene maps.

Using three separate data sets—combined, wildtype, and mutant—the 17 transcripts contained in Figure 5A were used to generate heatmaps (Figure 6). The heatmap generated from the combined data set showed two strong cis-eQTLs: *Tyrp1* on chromosome 4 and *Myo5a* on chromosome 9. Because *Tyrp1* expression levels within either the wildtype or mutant data sets have far less variability than in the combined data set, the strong cis-eQTL for *Tyrp1* was eliminated in panels B and C. In the wildtype data set, *Tyrp1* was the candidate gene for the major trans-eQTL for *Mitf, Dct,* and *Tyr*. *Myo5a* was a candidate gene for the trans-eQTL for *Tyrp1*, *Tyr*, *Si, Ednrb*, *Slc45a2*, Cbp/p300-interacting transactivator 1 (*Cited1*), and *Sema3c*. All of these, with the exception of *Sema3c*, are genes known to be involved with pigmentation. The heatmap generated using the mutant strains had minimal banding, revealing only a cis-eQTL on chromosome 9 at the position of *Myo5a*. Two of 17 cis-eQTLs were consistently significant across data sets: *Myo5a* on chromosome 9 and *Ednrb* on chromosome 14.

**Figure 7:**
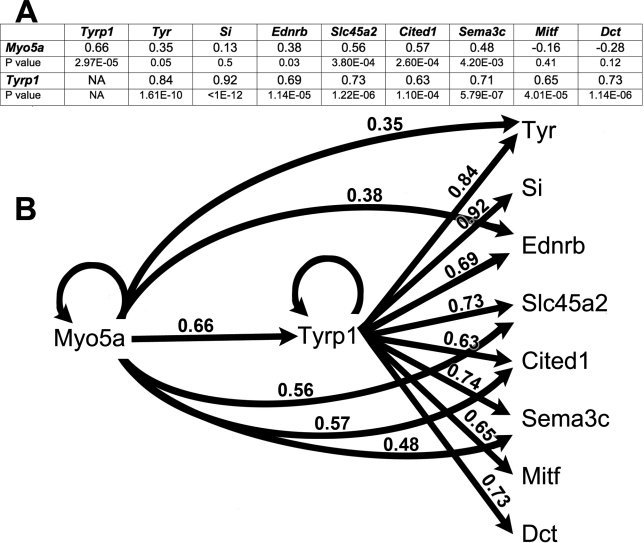
Partial correlation analysis and modeling of genes with expression in the eye that are modulated by *Tyrp1* or *Myo5a* in the heatmap. **A**: Summary table showing partial correlation coefficients and corresponding p values for all relationships. **B**: Composite model showing all Pearson correlation coefficients. Circular lines indicate cis-eQTLs. Linear lines indicate direct regulation of one gene by another. Edges that connect *Myo5a* directly to other genes/transcripts such as *Tyr* are labeled with the values of the partial correlation that removes the effects of variation in *Tyrp1*.

Because heatmap analyses suggested that *Myo5a* was a strong cis-eQTL that could be a master regulator of many pigmentation genes, we performed partial correlation analyses to determine the respective contributions of *Tyrp1* and *Myo5a* toward the regulation of the other genes in the networks and heatmap associated with wildtype *Tyrp1*. The outcomes are presented in Figure 7. These analyses and simple models suggest that *Tyrp1* is a cis-eQTL and therefore controls its own expression along with that of all other genes in the heatmap. It also suggests that *Myo5a* also controls its own level of expression along with that of all other genes with the exception of *Si*, *Mitf* and *Dct*. In almost all of these relations, *Tyrp1* has a stronger correlation with its targets than *Myo5a*. The exception is *Cited1*, over which both genes exert roughly equivalent control.

## Discussion

The pigmentation pattern of the iris is a complex genetic trait controlled by a network of genes whose coordinated regulation is poorly understood [32]. While several genes involved in this process are known—*Tyr*, *Tyrp1*, and *Dct*—it is unclear how these genes are co-regulated within the context of extended functional gene networks. In this study, we have examined expression patterns of normal and mutant *Tyrp1,* as well as other network-linked genes, to establish the regulatory mechanisms that underlie the wildtype iris pigmentation trait and to determine how this functional gene network is altered by mutation of one of its key gene components.

Much of what is known regarding pigmentation has arisen from studies of mouse coat color along with pigmentation defects in humans. Oculocutaneous albinism (OCA) exists as four types that are stratified based upon severity. Mutations in *Tyrp1* are causative for OCA3. The DBA/2J inbred mouse harbors the *Tyrp1^b^* mutation, one of the oldest known mutations and the first cloned locus [33]. Like OCA3, the brown phenotype is also due to mutations in *Tyrp1*. In mice, this mutation comprises a nonsynonymous exonic point mutation (C→Y at codon 110) [11]. Because it segregates in the BXD RI family of mice, we can use a systems genetics approach to define and identify the genes whose expression is linked to either wildtype or mutant *Tyrp1.* Our overall goal of this study was to construct the functional gene networks associated with *Tyrp1* and generate testable hypotheses about how these networks are co-regulated.

We have generated complete sequence data for D2 [34] and for all BXD strains we have high resolution SNP genetics maps, along with 500,000 genotypes that were generated using the mouse diversity array. In addition, we now have a database of ~4.8M SNPs, 500K indels, and thousands of inversions and copy number variants (CNVs) that are segregating among the BXD lines. Using these resources, we have readily confirmed the point mutation in exon 2 at codon 110 and also identified multiple intronic, exonic and 3′UTR SNPs. Consistent with the influences of many missense mutations, Tyrp1 protein was produced at a roughly equivalent amount in both whole eyes and irides of mice harboring the mutation.

The *Tyrp1^b^* mutation results in production of a gene product with an aberrant function and disruption of gene regulatory networks. To fully understand the genetic changes associated with *Tyrp1*, data for the RI strains were stratified into mutant and wildtype subsets, which were analyzed individually. Analysis of wildtype strains revealed a correlated network including a small set of genes with known relationships. In contrast, mutant strain analysis revealed significant loss of correlation to traditional genes and gain of correlation to genes with little or no functional relationship. This finding confirms that the mutation in *Tyrp1* directly affected the genetic networks associated with the gene, as well as the normal biologic functions of the protein. Thus, one of the major findings of our study is that a mutation in a single gene, such as *Tyrp1,* can significantly alter the functional associations of the mutant gene within the network required to maintain expression of a complex trait. Disruption of the network results in aberrant function of other genes not directly mutated.

Wildtype *Tyrp1* is associated with mesenchyme and melanin/pigmentation gene networks. Our gene ontology and network graphs show functional clusters of genes with expression levels that closely correlate with expression levels of either wildtype or mutant *Tyrp1*. In the subset of RI lines with wildtype *Tyrp1* the network of the genes with the highest correlation coefficients included many genes that function in mesenchyme or melanin/pigmentation (94%). Interestingly, the expression of wildtype *Tyrp1* was significantly correlated with six genes involved in mesenchymal cell development—these genes comprise 44% of the genes in the entire network. This is the first time that *Tyrp1* has been linked to mesenchymal development. Functionally, this is not entirely surprising because the uveal tract of the eye—iris, ciliary body and choroid—is highly pigmented and is derived of both neuroectodermal and mesenchymal origins.

Mutant *Tyrp1* is uncoupled from its normal gene networks. In contrast to wildtype *Tyrp1*, gene ontology and network graphs generated from the mutant database included very few genes involved in mesenchyme or melanin/pigmentation. Most of genes belonged to “other” categories. This demonstrates that the mutation in *Tyrp1* causes a shift to very different functional pathways in the eye. Previous studies have demonstrated that the *Tyrp1^b^* mutation causes an endoplasmic reticulum disease where the mutant *Tyrp1* affects the stability and function of other members of the melanogenic pathway [35]. Thus *Tyrp1* is a key player in both the genetic regulation and biochemical regulation of components of the melanogenic pathway and both functions are disrupted by mutation of this single gene.

Our data support the findings of other groups that document a role for mutant *Tyrp1* in ocular disease. Specifically, the iris disease found in D2 mice—due to mutations in both *Tyrp1* and *Gpnmb*—is not rescued by introgression of the mutant genes on to a B6 background. The iris pigmentation defect remains despite the gain of protection against an increased intraocular pressure. Moreover, the iris disease appears to be due to the formation of toxic products of the melanin pathway, as deficiency of TYR mitigated the degradation of the iris [36]. Another recent study has demonstrated that *Tyrp1* modifies the severity of the iris transillumination defect found in Lyst^bg-J^ mice. The *Tyrp1^b^* mutation is predicted to increase the iris defect by affecting lipid hydroperoxide levels [37].

Expression of *Tyrp1* is under its own control and is regulated upstream by *Myo5a*. Our mapping studies strongly suggest that *Tyrp1* controls its own expression. The LRS score for this cis-eQTL is 56.1 and is among the top 3.69% of all cis-eQTLs in the mouse genome. Composite interval mapping suggested that another locus on chromosome 9 at ~75 Mb was also responsible for controlling *Tyrp1* expression levels. This same relationship between *Tyrp1* and *Myo5a* was present repeatedly in our analyses. Specifically, in mice with wildtype *Tyrp1*, the expression levels of *Tyrp1* and *Myo5a* are highly correlated (p=3.0×10^−5^), *Myo5a* controls *Tyrp1* expression via heatmap analysis and partial correlation analysis suggests that *Myo5a* regulates the expression of many pigmentation genes both directly and indirectly through its control of *Tyrp1*. *Myo5a* also has a highly significant cis-eQTL with an LRS score of 155.3. Analysis of all 45,101 probes on the Affymetrix chip (M430V2) revealed that only 129 probes have cis-eQTLs of 155.3 or greater. *Myo5a* is therefore in the top 0.29% of all probes for the likelihood of its self-regulation. This strongly suggests that *Myo5a* is a cis-eQTL and likely functions in downstream regulation of many pigmentation genes.

Other studies [38,39] have shown that *Mitf*, a transcription factor, binds to the promoter regions of *Tyr*, *Tyrp1*, *Dct*, *Hif1a*, *Si*, *Ednrb*, and *Slc45a2*, thus regulating the expression of these genes. *Mitf* has been called a master regulator of melanocyte development [40]. Our analyses suggest that *Tyrp1*, with its upstream regulation by *Myo5a*, is an additional regulator of the melanin/pigmentation genes in the eye of BXD mice with wildtype *Tyrp1*. To evaluate genetic relationships of *Mitf* with other pigmentation genes, we performed a bioinformatics analysis of *Mitf* genetic regulation in the BXD strains with wildtype *Tyrp1*. We found that *Mitf* does not regulate its own expression and that it has very high expression levels. Moreover, the expression level of *Mitf* is highly correlated with the levels of many of the known pigmentation genes (data not shown). Based upon these data, it appears that *Tyrp1* and *Mitf* both regulate/modulate the expression of pigmentation genes in the eye.

In summary, our data suggest that wildtype *Tyrp1* functions within a gene network involved in melanin production, pigmentation and mesenchyme development and that it regulates its own expression. In addition, *Myo5a* is an upstream regulator of the wildtype *Tyrp1* gene, and in turn, *Tyrp1* provides downstream regulation of other pigmentation and mesenchyme-related genes. However, the mutation disrupts normal function of the gene and uncouples it from the tight network of genes in which *Tyrp1* normally interacts. *Myo5a* continues to regulate mutant *Tyrp1* expression*;* however, mutant *Tyrp1* no longer provides downstream regulation of the normal complement of other pigmentation and mesenchyme-related genes. Interestingly, the expression of mutant *Tyrp1* now co-varies with a different set of genes that are generally unrelated to pigmentation or melanin synthesis. We find no evidence that mutant *Tyrp1* regulates expression of any of these new co-variant genes, suggesting that the mutation alters the ability of the gene to regulate expression of other genes as well as its ability to produce protein that participates in pigmentation metabolism.

## Appendix

Appendix 1. List of SNPs within *Tyrp1*. To access the data, click or select the words “Appendix 1.” This will initiate the download of a compressed (pdf) archive that contains the file.

Appendix 2. WT data - *Tyrp1* correlates in enriched biologic process categories. To access the data, click or select the words “Appendix 2.” This will initiate the download of a compressed (pdf) archive that contains the file.

Appendix 3. Mutant data - *Tyrp1* correlates in enriched biologic process categories. To access the data, click or select the words “Appendix 3.” This will initiate the download of a compressed (pdf) archive that contains the file.

## References

[1] Wang N, Hebert D (2006). Tyrosinase maturation through the mammalian secretory pathway: bringing color to life.. Pigment Cell Res.

[2] Manga P, Kromberg J, Box N, Sturm R, Jenkins T, Ramsay M (1997). Rufous oculocutaneous albinism in southern African Blacks is caused by mutations in the TYRP1 gene.. Am J Hum Genet.

[3] Boissy RE, Zhao H, Oetting WS, Austin LM, Wildenberg SC, Boissy YL, Zhao Y, Sturm RA, Hearing VJ, King RA, Nordlund JJ (1996). Mutation in and lack of expression of tyrosinase-related protein-1 (TRP-1) in melanocytes from an individual with brown oculocutaneous albinism: a new subtype of albinism classified as 'OCA3'.. Am J Hum Genet.

[4] Rooryck C, Roudaut C, Robine E, Musebeck J, Arveiler B (2006). Oculocutaneous albinism with TYRP1 gene mutations in a Caucasian patient.. Pigment Cell Res.

[5] Chiang P-W, Spector E, Scheuerle A (2009). A case of Asian Indian OCA3 patient.. Am J Med Genet.

[6] Forshew T, Khaliq S, Tee L, Smith U, Johnson C, Mehdi S, Maher ER (2005). Identification of novel TYR and TYRP1 mutations in oculocutaneous albinism.. Clin Genet.

[7] King RA, Rich S (1986). Segregation analysis of brown oculocutaneous albinism.. Clin Genet.

[8] Wei A, Wang Y, Long Y, Wang Y, Guo X-J, Zhou Z, Zhu W, Liu J, Bian X, Lian S, Li W (2010). A Comprehensive Analysis Reveals Mutational Spectra and Common Alleles in Chinese Patients with Oculocutaneous Albinism.. J Invest Dermatol.

[9] Sturm RA, Frudakis TN (2004). Eye colour: portals into pigmentation genes and ancestry.. Trends Genet.

[10] Cohen T, Muller RM, Tomita Y, Shibahara S (1990). Nucleotide sequence of the cDNA encoding human tyrosinase-related protein.. Nucleic Acids Res.

[11] Zdarsky E, Favor J, Jackson I (1990). The Molecular Basis of brown, an Old Mouse Mutation, and of an Induced Revertant to Wild Type.. Genetics.

[12] Lamoreux M, Delmas V, Larue L, Bennett DC. The Colors of Mice: A Model Genetic Network. Hoboken, NJ: Wiley-Blackwell; 2010.

[13] Anderson MG, Smith RS, Hawes NL, Zabeleta A, Chang B, Wiggs JL, John SW (2002). Mutations in genes encoding melanosomal proteins cause pigmentary glaucoma in DBA/2J mice.. Nat Genet.

[14] Lynch S, Yanagi G (2002). DelbBono E, Wiggs JL. DNA sequence variants in the tyrosinase-related protein 1 (TYRP1) gene are not associated with human pigmentary glaucoma.. Mol Vis.

[15] Geisert EE, Lu L, Freeman-Anderson NE, Templeton JP, Nassr M, Wang X, Gu W, Jiao Y, Williams RW (2009). Gene expression in the mouse eye: an online resource for genetics using 103 strains of mice.. Mol Vis.

[16] Templeton JP, Nassr M, Vazques-Chona F, Freeman-Anderson NE, Orr WE, Williams RW, Geisert EE (2009). Differential response of C57BL/6J mouse and DBA/2J mouse to optic nerve crush.. BMC Neurosci.

[17] Lu H, Wang X, Pullen M, Guan H, Chen H, Sahu S, Zhang B, Chen H, Williams RW, Geisert EE, Lu L, Jablonski MM (2011). Genetic Dissection of the Gpnmb Network in the Eye.. Invest Ophthalmol Vis Sci.

[18] Jablonski MM, Freeman NE, Orr WE, Templeton JP, Lu L, Williams RW, Geisert EE (2011). Genetic Pathways Regulating Glutamate Levels in Retinal Müller Cells.. Neurochem Res.

[19] Whitney IE, Raven M, Lu L, Williams R, Reese BA (2011). QTL on Chromosome 10 Modulates Cone Photoreceptor Number in the Mouse Retina.. Invest Ophthalmol Vis Sci.

[20] Whitney IE, Raven M, Ciobanu D, Poché R, Ding Q, Elshatory Y, Gan L, Williams RW, Reese BE (2011). Genetic modulation of horizontal cell number in the mouse retina.. Proc Natl Acad Sci USA.

[21] Chesler EJ, Lu L, Shou S, Qu Y, Gu J, Wang J, Hsu HC, Mountz JD, Baldwin NE, Langston MA, Threadgill DW, Manly KF, Williams RW (2005). Complex trait analysis of gene expression uncovers polygenic and pleiotropic networks that modulate nervous system function.. Nat Genet.

[22] Chang B, Khanna H, Hawes N, Jimeno D, He S, Lillo C, Parapuram SK, Cheng H, Scott A, Hurd RE, Sayer JA, Otto EA, Attanasio M, O'Toole JF, Jin G, Shou C, Hildebrandt F, Williams DS, Heckenlively JR, Swaroop A (2006). In-Frame Deletion in a Novel Centrosomal/Ciliary Protein CEP290/NPHP6 Perturbs its Interaction with RPGR and Results in Early-Onset Retinal Degeneration in the rd16 mouse.. Hum Mol Genet.

[23] Hegmann JP, Possidente B (1981). Estimating genetic correlations from inbred strains.. Behav Genet.

[24] Zhang B, Schmoyer D, Kirov S, Snoddy J (2004). GOTree Machine (GOTM): a web-based platform for interpreting sets of interesting genes using Gene Ontology hierarchies.. BMC Bioinformatics.

[25] Benjamini Y, Hochberg Y (1995). Controlling the false discovery rate: a practical and powerful approach to multiple testing.. J R Stat Soc, B.

[26] Zhang B, Kirov S, Snoddy J (2005). WebGestalt: an integrated system for exploring gene sets in various biological contexts.. Nucleic Acids Res.

[27] Duncan D, Prodduturi N, Zhang B (2010). WebGestalt2: an updated and expanded version of the Web-based Gene Set Analysis Toolkit.. BMC Bioinformatics.

[28] de la Fuente A, Bing N, Hoeschele I, Mendes P (2004). Discovery of meaningful associations in genomic data using partial correlation coefficients.. Bioinformatics.

[29] Mozhui K, Ciobanu D, Schikorski T, Wang X, Lu L, Williams R (2008). Dissection of a QTL hotspot on mouse distal chromosome 1 that modulates neurobehavioral phenotypes and gene expression.. PLoS Genet.

[30] Jenkins NA, Copeland N, Taylor B, Lee B (1981). Diltue (d) coat colour mutation of DBA/2J mice is associated with the site of integration of an ecotropic MuLV genome.. Nature.

[31] Shibahara S, Tomita Y, Yoshizawa M, Shibata K, Tagami H (1992). Identification of mutations in the pigment cell-specific gene located at the brown locus in mouse.. Pigment Cell Res.

[32] Frudakis T, Thomas M, Gaskin Z, Venkateswarlu K, Chandra K, Ginjupalli S, Gunturi S, Natrajan S, Ponnuswamy VK, Ponnuswamy KN (2003). Sequences associated with human iris pigmentation.. Genetics.

[33] Smyth IM, Wilming L, Lee A, Taylor M, Gautier P, Barlow K, Wallis J, Martin S, Glithero R, Phillimore B, Pelan S, Andrew R, Holt K, Taylor R, McLaren S, Burton J, Bailey J, Sims S, Squares J, Plumb B, Joy A, Gibson R, Gilbert J, Hart E, Laird G, Loveland J, Mudge J, Steward C, Swarbreck D, Harrow J, North P, Leaves N, Greystrong J, Coppola M, Manjunath S, Campbell M, Smith M, Strachan G, Tofts C, Boal E, Cobley V, Hunter G, Kimberley C, Thomas D, Cave-Berry L, Weston P, Botcherby MR, White S, Edgar R, Cross SH, Irvani M, Hummerich H, Simpson EH, Johnson D, Hunsicker PR, Little PF, Hubbard T, Campbell RD, Rogers J, Jackson IJ (2006). Genomic anatomy of the Tyrp1 (brown) deletion complex.. Proc Natl Acad Sci USA.

[34] Wang X, Agarwala R, Capra J, Chen Z, Church D, Ciobanu DC (2010). High-throughput sequencing of the DBA/2J mouse genome.. BMC Bioinformatics.

[35] Toyofuku K, Wada I, Valencia J, Kushimoto T, Ferrans V, Hearing V (2001). Oculocutaneous albinism types 1 and 3 are ER retention diseases: mutation of tyrosinase or Tyrp1 can affect the processing of both mutant and wild-type proteins.. FASEB J.

[36] Anderson MG, Libby RT, Mao M, Cosma IM, Wilson LA, Smith RS, John SW (2006). Genetic context determines susceptibility to intraocular pressure elevation in a mouse pigmentary glaucoma.. BMC Biol.

[37] Trantow CM, Hedberg-Buenz A, Iwashita S, Moore SA, Anderson MG (2010). Elevated Oxidative Membrane Damage Associated with Genetic Modifiers of Lyst-Mutant Phenotypes.. PLoS Genet.

[38] Murisier F, Beermann F (2006). Genetics of pigment cells: lessons from the tyrosinase gene family.. Histol Histopathol.

[39] Hoek KS, Schlegel N, Eichhoff O, Widmer D, Praetorius C, Einarsson S, Valgeirsdottir S, Bergsteinsdottir K, Schepsky A, Dummer R, Steingrimsson E (2008). Novel MITF targets identified using a two-step DNA microarray strategy.. Pigment Cell Melanoma Res.

[40] Tachibana M (1997). Evidence to suggest that expression of MITF induces melanocyte differentiation and haploinsufficiency of MITF causes Waardenburg syndrome type 2A.. Pigment Cell Res.

